# Demography, commonly diagnosed disorders and mortality of guinea pigs under primary veterinary care in the UK in 2019—A VetCompass study

**DOI:** 10.1371/journal.pone.0299464

**Published:** 2024-03-27

**Authors:** Dan G. O’Neill, Jacques L. Taffinder, Dave C. Brodbelt, Vicki Baldrey

**Affiliations:** 1 Pathobiology and Population Sciences, The Royal Veterinary College, Hatfield, United Kingdom; 2 Clinical Science and Services, The Royal Veterinary College, Hatfield, United Kingdom; Universidade Federal de Minas Gerais, BRAZIL

## Abstract

**Introduction:**

Guinea pigs are popular as domestic pets but there is limited information on the health of the wider pet population. This study aimed to report demography, commonly diagnosed disorders and mortality of guinea pigs under UK primary veterinary care.

**Methods:**

Diagnosis and mortality information on guinea pigs was extracted from anonymised UK primary-care clinical records in VetCompass.

**Results:**

From 51,622 guinea pigs under primary veterinary care during 2019, a specific breed was not recorded in 50,098 (97.05%). Of guinea pigs with information recorded, 23,206 (47.33%) were female and 25,828 (52.67%) were male. There were 1,020 (2.08%) neutered and 48,014 (97.92%) entire. Median adult bodyweight overall was 1.05kg (interquartile range [IQR] 0.90–1.19, range 0.40–2.66). From a random sample of 3,785/51,622 (7.33%) guinea pigs, the most prevalent disorders were overgrown nail(s) (n = 1,005, 26.55%, 95% confidence interval [CI] 25.15–27.99), dermatophytosis (228, 6.02%, 95% CI 5.29–6.83) and corneal ulceration (189, 4.99%, 95% CI 4.32–5.74). Among the 30 most common disorders, females showed predisposition for 3 disorders and males showed predisposition for 5 disorders. The disorder with the youngest age of affected animals was dermatophytosis (1.11 years) while weight loss had the oldest age of affected animals (4.64 years). From 757 recorded deaths, the median age at death overall was 4.03 years (IQR 2.56–5.44, range 0.17–10.00). Among deaths with a recorded cause, the most common causes of death were anorexia (n = 82, 13.87%, 95% CI 11.19–16.93), collapsed (58, 9.81%, 95% CI 7.54–12.50) and peri-anaesthetic death (20, 3.38%, 95% CI 2.08–5.18).

**Conclusions:**

These results can assist veterinarians and owners by providing demographic, disorder and mortality benchmarks that support improved clinical care and welfare outcomes in guinea pigs. Many common disorders in guinea pigs were husbandry related.

## Introduction

Guinea pigs are rodents in the *Caviidae* family. The domestic guinea pig, *Cavia porcellus*, was introduced into Europe over 500 years ago and is now a popular domestic pet, owing to public perceptions of docile nature, lively personality, and perceived ease of care [1]. However, despite their popularity, the volume of robust information available on the demography, disorders and mortality of guinea pigs kept as domestic pets is very limited compared with other commonly owned pet species such as dogs and cats [2], with much of the available information being extrapolated from research on laboratory guinea pigs. Further understanding of the demography of the pet guinea pig population and the most common clinical presentations to primary-care practice would help improve veterinary and owner awareness of common disorders and to manage wider expectations regarding longevity and common health problems. Enhanced information relating to guinea pigs could also inform improved curriculum design in undergraduate veterinary and biological sciences education as well as improved continuing professional development (CPD) for veterinary professionals.

The UK pet guinea pig population is estimated at approximately 900,000 in 2022, with 1.3% of UK households owning at least one guinea pig [3]. The British Cavy Council formally recognises and has published breed standards on 28 distinct breeds of guinea pig, with new breeds regularly added [4]. The most common guinea pig breeds kept as pets in the USA are the American (also known as the English or Short-haired guinea pig), Peruvian and Abyssinian [5]. Breed frequency in guinea pigs is not well reported in the UK, with a recent UK survey reporting that 39.8% of owners were unaware what breed their own guinea pig was [6].

There is limited literature on overall health in guinea pigs, with most published information comprising textbook descriptions drafted by experienced practitioners, individual case reports, small case series or prevalence studies of a particular condition [1, 7–12]. A retrospective study of guinea pigs presenting to a specialist exotics clinic in Czech Republic reported dental disease (36.3%), skin disease (33.1%) and ovarian cystic disease (21.9% of females) as the most common presentations [13]. A review of skin disease of guinea pigs at a specialist exotics clinic in the USA reported pododermatitis and louse infestation (pediculosis) as the most common dermatological problems observed [14]. A UK survey of veterinarians’ perceptions of disorder presentations in guinea pigs cited skin, non-specific (e.g. inappetence) and dental disease as the most common [15]. Lifespan in guinea pigs is reported from 4–9 years, or 4–7 years for indoor pets [16], although survival to over 20 years is also reported [6]. A study of the clinical confidence of veterinarians in general practice in treating rabbits, guinea pigs and exotic pets identified a relative lack of knowledge of, and confidence in, diagnosing and treating these species compared to treating dogs and cats [17]. Better understanding of the demographics and disorder frequencies of guinea pigs under primary veterinary care could contribute to improved welfare of pet guinea pigs by increasing veterinarian understanding of disease frequency and by providing owners with an evidence base on likely disorder risk.

To fill these data gaps on the health of guinea pigs kept as domestic pets in the UK, this VetCompass study aimed to apply a practice-based research approach to report the demography, commonly diagnosed disorders and causes of mortality of guinea pigs under primary veterinary care in the UK [18]. By exploring real-world anonymised clinical data that are representative of typical UK primary care practices, the study aimed for results that can be generalised to the wider population of UK primary care practice. The study was specifically interested in comparing differences in disease prevalence between the sexes and exploring age-related disease. The results could provide an improved evidence base for disorder prioritisation in guinea pigs that could assist veterinary professionals and owners to identify opportunities to improve the care and welfare of pet guinea pigs overall.

## Methods

The study included all guinea pigs under primary veterinary care within VetCompass in 2019. Being ‘under veterinary care’ required ≥ 1 electronic patient record (EPR) during 2019 that included at least one bodyweight, free-text clinical note or treatment record. VetCompass is a research programme that shares anonymised clinical records from primary-care veterinary practices in the UK [18]. Further information on the specialisms, experience or advanced qualifications of the veterinary professionals within these practices was not available for the current study. The data fields available for the current study included a unique animal ID with associated metadata as time-invariant variables (species, breed, sex, neuter and date of birth) and time-varying variables (bodyweight, free-form text clinical notes and treatments with associated dates). The design and analytic plans for the current study were deliberately aligned with previous VetCompass species-based studies to facilitate comparison between species [19–21]. Ethics approval was obtained from the Royal Veterinary College Ethics and Welfare Committee (reference number SR2018-1652).

A retrospective cohort study design that followed the entire available clinical history of each guinea pig was used to estimate the prevalence of the most commonly diagnosed disorders for guinea pigs under veterinary care during 2019. Sample size calculation using OpenEpi software (www.openepi.com) showed that a sample of 3,493 guinea pigs was needed from a population of 51,622 guinea pigs to estimate the prevalence of a disorder occurring in 2.5% of guinea pigs to within an absolute 0.5% margin of error.

All available clinical records from a randomly selected subset of guinea pigs were manually reviewed and all disorder events at any date in the cohort data were followed over time within all available clinical records to determine the most definitive diagnosis term recorded, as previously described [19]. Randomisation used the RAND (transact-SQL) function within SQL Server. All diagnoses were made by veterinary surgeons registered with the Royal College of Veterinary Surgeons (www.rcvs.org.uk). The precise criteria used to determine each final diagnosis was at the clinical discretion of the attending veterinary surgeon (e.g. laboratory testing, necropsy, advanced imaging, referral) and information on the precise diagnostic testing and processes for each diagnosis in the current study was not extracted. The diagnostic processes and terms applied in the current study reflect those typical of UK primary veterinary care and no effort was made to select and include only those cases worked up to a higher standard or to referral level criteria. Incident and pre-existing presentations were not differentiated. Recurring ongoing conditions (e.g., dental overgrowth) were recorded only once. Clinical conditions that were not recorded with a biomedical diagnostic term were extracted using the first recorded presenting sign term (e.g., ‘lethargy’) as previously described [21]. Mortality data were extracted for all deaths recorded at any date in the available records. These data included the final biomedical issue that triggered the decision to euthanase or that led to the unassisted death (hereinafter called the cause of death), the date of the death, the process by which the death occurred (hereinafter called the mechanism of death and classified as either euthanasia, unassisted death, unrecorded) and the method of body disposal (communal cremation, individual cremation, burial). All extracted diagnostic terms were retrospectively mapped to both precise and grouped levels of diagnostic precision as described previously [21] (S1 Table). Precise-level terms provided disorder information to the highest level of diagnostic precision available within the clinical notes (e.g., *cystitis* would remain as *cystitis*) while disorder groups provided information at a more general level of diagnostic precision (e.g., *cystitis* would map to *urinary system disorder*).

Data checking and cleaning used Excel (Microsoft Office Excel 2013, Microsoft Corp.) and analysis used Stata Version 16 (Stata Corporation). The breed information recorded in the EPR was mapped to a VetCompass list of guinea pig breeds. All terms entered by the veterinary practices as describing a specific breed were accepted as a unique breed. Adult bodyweight (kg) described the median of all recorded bodyweights recorded for each guinea pig over 6 months of age. The age (years) for each guinea pig was calculated at 31 December 2019.Descriptive results for guinea pigs under veterinary care in 2019 were reported for breed, sex, neuter status and adult (> 6 months) bodyweight. Disorder prevalence values described the percentage of guinea pigs recorded with a specific diagnosis at least once at any date during the available clinical records for each guinea pig. The 95% confidence intervals (CI) estimates were calculated using the Clopper-Pearson method based directly on the binomial distribution. Multivariable binary logistic regression modelling was used to estimate the odds in male relative to female for common causes of morbidity and mortality. A separate model was built for each disorder that included a standard bank of covariables alongside sex to account for confounding factors in morbidity (neuter, age, veterinary group) and mortality (neuter, veterinary group) [22, 23]. The authors applied an ‘information theory’ approach to decide on covariables to include in these standard models [24]. Results from each regression model were reported only for associations with sex (*a priori* interest). The median age (years) and median adult bodyweight (kg) were also reported for each common cause of morbidity and mortality. The one-sample test of proportions was used to assess the proportion of male guinea pigs in the overall study population against a hypothesised 50% population proportion. The chi^2^ test and Mann-Whitney U test were used for additional comparisons as appropriate [25]. Statistical significance was set at the 5% level.

## Results

### Demography

The study population included 51,622 guinea pigs under primary veterinary care at 1,003 veterinary practices that were part of 6 veterinary groups across the UK participating in VetCompass during 2019. Among these guinea pigs, a specific breed was not recorded in 50,098 (97.05%), with the remaining guinea pigs described across 22 breed names (Table 1). There were 49,034/51,622 (94.99%) guinea pigs with information on sex recorded. Of these, 23,206 (47.33%) were female and 25,828 (52.67%) were male. The proportion of males in the population was significantly higher than a hypothesised 50% male proportion (P < 0.001). Overall, there were 1,020 (2.08%) guinea pigs recorded as neutered and 48,014 (97.92%) recorded as entire. Males (838/25,828, 3.24%) were more likely to be neutered than females (182/23,206, 0.78%) (P < 0.001). The median adult bodyweight of guinea pigs overall was 1.05kg (N = 22,912, interquartile range [IQR] 0.90–1.19, range 0.40–2.66). The median adult bodyweight of males (N = 10,008, 1.10kg, IQR 0.94–1.23, range 0.40–2.66) was statistically significantly heavier than for females (N = 12,267, 1.00kg, IQR 0.88–1.12, range 0.40–2.27) (P < 0.001). (Table 1). The median age on 31 December, 2019 of guinea pigs overall was 2.21 years (N = 43,488, IQR 1.09–3.99, range 0.00–10.99). The median age of females (N = 19,305, 2.33 years, IQR 1.09–4.24, range 0.00–10.99) was statistically significantly older than for males (N = 22,782, 2.14 years, IQR 1.11–3.83, range 0.00–10.99) (P < 0.001). (Table 1).

**Table 1:** Count, bodyweight (kg) and age (years on 31 December 2019) of guinea pig breeds under primary veterinary care at practices participating in the VetCompass programme in the UK from January 1^st^ to December 31^st^, 2019. n = 51,622.

Breed	No.	%	Median adult bodyweight kg	Median age years
Breed unrecorded	50,098	97.05	1.05	2.20
Abyssinian	661	1.28	1.07	2.39
Teddy	135	0.26	0.99	2.46
Crested	128	0.25	1.01	1.76
Peruvian	113	0.22	1.00	2.72
Rex	113	0.22	1.04	2.11
American crested	78	0.15	1.11	2.08
Dutch	67	0.13	1.14	2.51
Himalayan	51	0.10	1.10	2.89
Coronet	31	0.06	0.94	2.92
Texel	27	0.05	1.00	2.28
English Crested	25	0.05	1.08	1.64
Silky	25	0.05	1.10	2.31
Alpaca	19	0.04	0.86	2.45
Skinny	10	0.02	0.81	2.50
Rosette	8	0.02	1.19	4.83
American	7	0.01	1.38	1.14
English Merino	7	0.01	0.75	0.98
Ridgeback	7	0.01	1.00	1.53
Satin	7	0.01	1.23	2.22
Californian	2	0.00	1.07	1.14
Swiss	2	0.00	Unrecorded	5.78
Exotic	1	0.00	1.23	2.90

### Disorder prevalence

The EPRs of a random sample of 3,785/51,622 (7.33%) guinea pigs were manually examined to extract information on all recorded disorders at any date in the available clinical records. At least one disorder was recorded for 3,288/3,785 (86.87%) guinea pigs. The remaining 13.13% did not have any disorder recorded. There was no evidence that the proportion of guinea pigs having at least one disorder recorded differed between females (1,477/1,705, 86.63%) and males 1,659/1,913, 86.72%) (P = 0.933). The median disorder count per guinea pig was 1 disorder (IQR 1–2, range 0–19). There was no evidence that median disorder count varied between females (n = 1,705, median 1, IQR 1–2, range 0–14) and males (n = 1,913, median 1, IQR 1–2, range 0–19) (P = 0.708).

There were 5,672 unique disorder events recorded across the 3,785 guinea pigs, spanning 324 separate precise-level disorder terms. The most prevalent precise-level disorders recorded across all guinea pigs were overgrown nail(s) (n = 1,005, 26.55%, 95% confidence interval [CI] 25.15–27.99), dermatophytosis (228, 6.02%, 95% CI 5.29–6.83) and corneal ulceration (189, 4.99%, 95% CI 4.32–5.74). The odds ratios differed statistically significantly between females and males for 8/30 precise-level disorders, with females having higher odds of three (corneal ulceration, foreign body, abdominal mass) and males having higher odds of five (anorexia, bite injury, overgrown incisor(s), dental disease, wound). The median age of affected guinea pigs varied by disorder from dermatophytosis (1.11 years) to weight loss (4.64 years). The median adult bodyweight varied by disorder from 0.84kg (thin) to 1.19kg (obesity) (Table 2).

**Table 2:** Prevalence of the 30 most common disorders at a *precise-level of diagnostic precision* recorded in guinea pigs (n = 3,785) under primary-care veterinary care at UK practices participating in the VetCompass™ programme from January 1^st^ to December 31^st^, 2019. The odds of the disorder in males compared with females is reported after accounting for confounding from neuter status, age and veterinary group attended. The median age and bodyweight for affected guinea pigs is also shown. Statistically significant results are shown in bolded text. *CI confidence interval.

Precise-level disorder	No.	%	95% CI*	Male: female odds ratio	95% CI*	Median age (years)	Median bodyweight (kg)
Overgrown nail(s)	1005	26.55	25.15–27.99	0.91	0.78–1.06	2.53	1.10
Dermatophytosis	228	6.02	5.29–6.83	1.04	0.74–1.46	1.11	1.00
Corneal ulceration	189	4.99	4.32–5.74	**0.61**	0.44–0.85	2.79	1.08
Diagnosis not completed	166	4.39	3.76–5.09	0.84	0.59–1.20	4.52	1.03
Anorexia	153	4.04	3.44–4.72	**1.62**	1.12–2.33	4.05	1.00
Abscess	152	4.02	3.41–4.69	0.72	0.51–1.02	3.34	1.04
Conjunctivitis	152	4.02	3.41–4.69	0.71	0.48–1.05	1.18	1.10
Upper respiratory tract infection	142	3.75	3.17–4.41	1.05	0.73–1.51	3.31	1.03
Mite infestation	122	3.22	2.68–3.84	1.13	0.76–1.67	2.22	1.06
Bite injury	121	3.20	2.66–3.81	**3.57**	2.12–6.01	1.71	1.04
Foreign body	108	2.85	2.35–3.43	**0.59**	0.38–0.91	2.73	1.13
Haircoat disorder	92	2.43	1.96–2.97	1.47	0.93–2.33	2.72	1.11
Obesity	81	2.14	1.70–2.65	0.86	0.54–1.35	2.95	1.19
Ocular discharge	81	2.14	1.70–2.65	1.43	0.82–2.47	3.34	1.04
Thin	77	2.03	1.61–2.54	1.55	0.93–2.57	3.74	0.84
Nasal discharge	76	2.01	1.59–2.51	1.03	0.59–1.78	1.49	1.09
Overgrown incisor(s)	76	2.01	1.59–2.51	**1.92**	1.15–3.20	4.01	1.00
Skin cyst	74	1.96	1.54–2.45	0.86	0.52–1.40	4.20	1.16
Collapsed	72	1.90	1.49–2.39	0.94	0.56–1.58	3.81	1.00
Ovarian disorder (female only)	64	3.75	2.90–4.77	~		4.62	1.04
Weight loss	67	1.77	1.37–2.24	1.41	0.83–2.41	4.64	0.94
Lung disorder	58	1.53	1.17–1.98	1.55	0.84–2.84	3.09	1.03
Haematuria	55	1.45	1.10–1.89	0.62	0.35–1.08	3.75	1.07
Pododermatitis	55	1.45	1.10–1.89	0.82	0.46–1.47	4.11	0.97
Abdominal distension	54	1.43	1.07–1.86	1.03	0.57–1.88	3.65	1.10
Overgrown molar(s)	54	1.43	1.07–1.86	1.23	0.69–2.19	3.78	0.95
Abdominal mass	51	1.35	1.00–1.77	**0.48**	0.27–0.88	4.63	1.01
Dental disease	50	1.32	0.98–1.74	**2.45**	1.25–4.81	3.64	1.02
Wound	50	1.32	0.98–1.74	**2.06**	1.04–4.07	1.62	1.15
Alopecia	49	1.29	0.96–1.71	0.78	0.42–1.44	3.78	1.06

All disorders across the guinea pigs were categorised into 58 grouped disorder terms. The most prevalent grouped-level disorders recorded across all guinea pigs were claw/nail disorder (n = 1,014, 26.79%, 95% CI 25.38–28.23), skin disorder (592, 15.64%, 95% CI 14.50–16.84), ophthalmological disorder (517, 13.66%, 95% CI 12.58–14.79) and upper respiratory tract disorder (289, 7.64%, 95% CI 6.81–8.53). The odds ratios differed statistically significantly between females and males for 8/20 grouped-level disorders with females having higher odds of four (mass, urinary system disorder, neoplasia, foreign body) and males having higher odds of four (traumatic injury, lower respiratory tract disorder, dental disorder, appetite disorder). The median age of affected guinea pigs by disorder varied from 1.63 years (traumatic injury) to 4.60 years (neoplasia). The median adult bodyweight varied by disorder from 0.88kg (underweight) to 1.19kg (obesity) (Table 3).

**Table 3:** Prevalence of the 20 most common disorders at a *grouped-level of diagnostic precision* recorded in guinea pigs (n = 3,785) under primary-care veterinary care at UK practices participating in the VetCompass programme from January 1^st^ to December 31^st^, 2019. The odds of the disorder in males compared with females is reported after accounting for confounding from neuter status, age and veterinary group attended. The median age and bodyweight for affected guinea pigs is also shown. Statistically significant results are shown in bolded text. *CI confidence interval.

Grouped-level disorder	No.	%	95% CI*	Male: female odds ratio	95% CI*	Median age (years)	Median bodyweight (kg)
Claw/nail disorder	1014	26.79	25.38–28.23	0.90	0.77–1.06	2.53	1.10
Skin disorder	592	15.64	14.50–16.84	1.02	0.83–1.25	2.14	1.07
Ophthalmological disorder	517	13.66	12.58–14.79	0.88	0.71–1.08	2.62	1.08
Upper respiratory tract disorder	289	7.64	6.81–8.53	1.10	0.84–1.43	2.55	1.06
Mass	224	5.92	5.19–6.72	**0.69**	0.52–0.93	4.22	1.10
Traumatic injury	221	5.84	5.11–6.63	**2.42**	1.72–3.41	1.63	1.05
Parasite infestation	219	5.79	5.06–6.58	1.11	0.82–1.49	2.36	1.05
Lower respiratory tract disorder	192	5.07	4.40–5.82	**1.80**	1.29–2.51	3.56	1.05
Urinary system disorder	191	5.05	4.37–5.79	**0.67**	0.49–0.92	4.13	1.07
Dental disorder	188	4.97	4.30–5.71	**1.97**	1.41–2.76	3.82	1.02
Appetite disorder	169	4.46	3.83–5.17	**1.60**	1.13–2.27	4.05	1.00
Diagnosis not completed	166	4.39	3.76–5.09	0.84	0.59–1.20	4.52	1.03
Abscess	153	4.04	3.44–4.72	0.71	0.51–1.01	3.34	1.04
Neoplasia	148	3.91	3.32–4.58	**0.68**	0.47–0.97	4.60	1.09
Underweight	137	3.62	3.05–4.26	1.45	0.99–2.13	4.33	0.88
Enteropathy	116	3.06	2.54–3.66	1.44	0.96–2.17	3.43	1.01
Foreign body	108	2.85	2.35–3.43	**0.59**	0.38–0.91	2.73	1.13
Musculoskeletal disorder	85	2.25	1.80–2.77	1.22	0.77–1.95	3.91	1.12
Female reproductive disorder (female only)	77	4.52	3.58–5.61	~		4.38	1.04
Obesity	81	2.14	1.70–2.65	0.86	0.54–1.35	2.95	1.19

### Mortality

There were 757 deaths recorded among the 3,785 (20.00%) guinea pigs. Information on the age at death was available for 674/757 (89.04%) of deaths. The median age at death overall was 4.03 years (IQR 2.56–5.44, range 0.17–10.00). The median age at death in females (4.58 years, IQR 3.00–5.81, range 0.25–8.25) was statistically older than males (3.74, IQR 2.51–5.00, 0.17–10.00) (P < 0.001). The mechanism of death was recorded for 694/757 (91.68%) of deaths. Among deaths with the mechanism recorded, 512 (73.78%) deaths involved euthanasia and 182 (26.22%) were unassisted deaths. Proportional death by euthanasia did not differ statistically between females (218/305, 71.48%) and males (276/365, 75.62%) (P = 0.225). The median age at death for deaths by euthanasia (4.33 years, 2.85–5.53, 0.17–10.00) was statistically older than for unassisted deaths (3.33 years, 2.03–4.93, 0.17–8.04) (P < 0.001). The owners dealt with disposal of the body for burial in 376/757 (49.67%) deaths while the veterinary clinic dealt with disposal for 381/757 (50.33%) deaths. Of these veterinary disposals, 275/381 (72.18%) involved communal cremation while 106/381 (27.82%) involved individual cremation with ashes returned to the owner.

There were 131 precise-level disorder terms used to describe the cause of death across the 757 deaths in the study. Among 591/757 (78.07%) deaths with a recorded cause of death, the most common causes of death at a precise-level were anorexia (n = 82, 13.87%, 95% CI 11.19–16.93), collapsed (58, 9.81%, 95% CI 7.54–12.50) and peri-anaesthetic death (20, 3.38%, 2.08–5.18). Neoplasia was the only precise-level cause of death that varied statistically between the sexes, showing 0.13 times the odds (95% CI 0.02–0.78) in males compared with females. The median age at death across the most common causes of death at a precise-level varied from 2.90 years (lung disorder) to 6.16 years (haematuria). The median adult bodyweight varied from 0.80kg (dental disease) to 1.14kg (paresis/paralysis) (Table 4).

**Table 4:** Proportional mortality from the 20 most common disorders at a *precise-level of diagnostic precision* recorded in guinea pigs (n = 757) under primary-care veterinary care at UK practices participating in the VetCompass programme from January 1^st^ to December 31^st^, 2019. The odds of the disorder in males compared with females is reported after accounting for confounding from neuter status and veterinary group attended. The median age at death, adult bodyweight and proportion of deaths with a recorded mechanism that involved euthanasia for each cause of death is also shown. Statistically significant results are shown in bolded text. *CI confidence interval.

Precise-level disorder	No.	Proportional mortality (n = 757)	Proportional mortality (n = 591)	95% CI*	Male: female odds ratio	95% CI*	Median age at death (years)	Median bodyweight (kg)	Proportional euthanasia
Diagnosis not completed	166	21.93							
Anorexia	82	10.83	13.87	11.19–16.93	1.64	0.99–2.73	4.54	1.01	83.75
Collapsed	58	7.66	9.81	7.54–12.50	0.81	0.46–1.42	3.80	0.95	86.21
Peri-anaesthetic death	20	2.64	3.38	2.08–5.18	1.00	0.39–2.57	3.61	1.10	0.00
Abdominal distension	18	2.38	3.05	1.81–4.77	2.17	0.75–6.31	3.95	0.99	72.22
Dyspnoea	18	2.38	3.05	1.81–4.77	0.77	0.27–2.20	3.63	1.04	72.22
Poor quality of life	18	2.38	3.05	1.81–4.77	0.73	0.28–1.88	4.76	1.01	94.44
Abdominal mass	17	2.25	2.88	1.68–4.57	0.49	0.18–1.33	4.62	1.04	94.12
Respiratory distress	17	2.25	2.88	1.68–4.57	1.52	0.55–4.19	5.86	1.00	64.71
Weight loss	17	2.25	2.88	1.68–4.57	1.20	0.44–3.23	6.04	0.89	81.25
Urolithiasis	14	1.85	2.37	1.30–3.94	1.03	0.35–3.08	4.44	1.05	92.86
Lung disorder	13	1.72	2.20	1.18–3.73	1.62	0.46–5.69	2.90	0.96	69.23
Paresis/paralysis	13	1.72	2.20	1.18–3.73	0.87	0.28–2.66	5.15	1.14	100.00
Dental disease	12	1.59	2.03	1.05–3.52	4.29	0.91–20.14	3.03	0.80	91.67
Abscess	11	1.45	1.86	0.93–3.31	1.14	0.31–4.22	5.13	1.07	100.00
Neoplasia	11	1.45	1.86	0.93–3.31	**0.13**	0.02–0.78	5.68	0.95	100.00
Pneumonia	11	1.45	1.86	0.93–3.31	1.39	0.40–4.86	3.08	1.09	90.91
Upper respiratory tract infection	11	1.45	1.86	0.93–3.31	0.79	0.22–2.80	3.84	0.94	10.00
Gastrointestinal stasis	8	1.06	1.35	0.59–2.65	~		3.52	1.07	57.14%
Haematuria	7	0.92	1.18	0.48–2.43	0.12	0.01–1.05	6.16	0.93	85.71

Among deaths with a recorded cause, the most common causes of death at a grouped level of precision were appetite disorder (n = 84, 14.21%, 95% CI 11.5–17.29), lower respiratory tract disorder (63, 10.66%, 95% CI 8.29–13.43) and collapsed (60, 10.15%, 7.84–12.87). Neoplasia was the only grouped-level cause of death that varied statistically between the sexes, showing 0.44 times the odds (95% CI 0.20–0.93) in males compared with females. The median age at death across the most common causes of death at a grouped-level varied from 3.06 years (complication associated with clinical care) to 6.04 years (thin). The median adult bodyweight varied from 0.89kg (thin) to 1.07kg (complication associated with clinical care) (Table 5).

**Table 5:** Proportional mortality from the 20 most common disorders at a *grouped-level of diagnostic precision* recorded in guinea pigs (n = 757) under primary-care veterinary care at UK practices participating in the VetCompass programme from January 1^st^ to December 31^st^, 2019. The odds of the disorder in males compared with females is reported after accounting for confounding from neuter status and veterinary group attended. The median age at death, adult bodyweight and proportion of deaths with a recorded mechanism that involved euthanasia for each cause of death is also shown. Statistically significant results are shown in bolded text. *CI confidence interval.

Grouped-level disorder	No.	Proportional mortality (n = 757)	Proportional mortality (n = 591)	95% CI*	Male: female odds ratio	95% CI*	Median age at death (years)	Median bodyweight (kg)	Proportional euthanasia
Diagnosis not completed	166	21.93							
Appetite disorder	84	11.10	14.21	11.5–17.29	1.61	0.97–2.66	4.54	1.01	82.93
Lower respiratory tract disorder	63	8.32	10.66	8.29–13.43	1.44	0.82–2.55	3.56	1.04	73.02
Collapsed	60	7.93	10.15	7.84–12.87	0.74	0.43–1.30	3.79	0.97	83.33
Mass	42	5.55	7.11	5.17–9.48	0.55	0.29–1.04	4.53	1.06	95.24
Neoplasia	35	4.62	5.92	4.16–8.14	**0.44**	0.20–0.93	5.61	0.99	94.29
Urinary system disorder	31	4.10	5.25	3.59–7.36	0.69	0.33–1.45	4.71	1.03	93.55
Complication associated with clinical care	26	3.43	4.40	2.89–6.38	0.94	0.41–2.14	3.06	1.07	19.23
Enteropathy	26	3.43	4.40	2.89–6.38	1.65	0.69–3.92	3.52	0.97	70.83
Abdominal disease	22	2.91	3.72	2.35–5.58	2.24	0.84–5.97	4.60	1.02	68.18
Dental disorder	22	2.91	3.72	2.35–5.58	2.60	0.93–7.27	3.63	0.92	86.36
Brain disorder	21	2.77	3.55	2.21–5.38	0.59	0.24–1.45	4.80	0.93	100.00
Spinal cord disorder	21	2.77	3.55	2.21–5.38	1.25	0.50–3.11	3.72	1.04	100.00
Poor quality of life	18	2.38	3.05	1.81–4.77	0.73	0.28–1.88	4.76	1.01	94.44
Thin	17	2.25	2.88	1.68–4.57	1.20	0.44–3.23	6.04	0.89	81.25

## Discussion

This study is the largest investigation of guinea pigs under primary veterinary care to date, and identifies overgrown nails, dermatophytosis and corneal ulceration as the most common specific diagnoses in pet guinea pigs presenting to primary-care practice in the UK. At a disorder group level, claw/nail, skin and ophthalmic disorders were the most prevalent. Males were predisposed to anorexia, bite injury, haircoat disorders and overgrown incisors. Females were predisposed to corneal ulceration, conjunctivitis, foreign bodies, and ovarian disorders. The most common specific causes of death in guinea pigs were ‘disorder not diagnosed’, anorexia and collapse. At a disorder group level, the most common causes of death were ‘disorder not diagnosed’, appetite disorder and lower respiratory tract disorder. At both the precise and grouped levels of diagnosis, females showed predisposition to death from neoplasia compared to males. These findings contribute to filling the data gaps on the demography, disorders and mortality of guinea pigs kept as domestic pets in the UK and can provide an evidence base to support widespread efforts to improve the welfare of these animals [26, 27].

### Demography

Overall, 97.05% of the guinea pigs in the current study did not have a formal breed name assigned. The lack of recording of a formal specific breed term for most guinea pigs in the current study suggests that the majority of guinea pigs in the UK are not a specific ‘pure’ breed but could also reflect generally low levels of recognition of specific breeds by owners or veterinary personnel, or even that guinea pig owners and/or veterinarians are unconcerned by breed status, or at least less concerned than for other domestic pets such as dogs. It is also possible that many guinea pigs are purchased from pet shops where limited information on the breed may have been provided. In a UK survey of 4,590 guinea pig owners, 39.8% reported not knowing the breed of their pet [6]. This limited awareness or recognition of breed status in guinea pigs is consistent with previous retrospective studies where breed in guinea pigs has either not been recorded or discussed [13, 28], or poorly reported in the data [14, 29]. In general, bodyweight was highly consistent across the breeds recorded in our study, suggesting limited selection towards extreme conformation as yet in guinea pigs, in contrast to increasing moves towards extreme conformations in pet rabbit breeds [30]. Indeed, much of the morphological differences used to drive breed formation for guinea pigs has traditionally focused on differential hair coat structure, texture, colour and length [5] rather than the exaggerated body morphology seen with some canine breeds [23]. Breed status may therefore have less clinical significance in guinea pigs compared to other common companion species. The satin coat type of guinea pig has a genetic mutation which leads to progressive renal disease and associated bone disease [31]. A prospective study of ocular disease in 1000 guinea pigs identified purebred status as a significant factor, with outbred pet guinea pigs having a higher prevalence of ocular lesions than pedigree show animals [32]. This is interesting as it runs counter to widely held beliefs that inbreeding is a risk factor for disease while outbreeding is often considered to reduce disease risk [33]. That study by Williams and Sullivan [32] also found that the Texel breed of guinea pig was prone to ocular surface irritation by trichiasis. The complex influence of breed on disease prevalence in guinea pigs therefore warrants further investigation.

The proportion of all guinea pigs presented to primary-care veterinary practices was significantly higher for males than for females, consistent with previous studies [13, 14] although this may reflect the demographic of the underlying pet population [6] rather than a truly increased disease prevalence in males. Just 2.08% of guinea pigs were recorded as neutered in our study, with males significantly more likely to be neutered than females. This is lower than the 10.9% of guinea pigs reported as neutered in an owner survey [6] although that study did agree with our finding that significantly more males were neutered than females. As an owner questionnaire distributed through guinea pig forums and social media groups, that questionnaire study may have been biased towards owners with a greater investment in the care of their guinea pig which might explain the higher proportional neutering, although the methodology did also try to engage less enthusiastic owners by using leaflets, posters and a prize draw as an incentive. Low levels of neutering may also reflect differential concerns about anaesthetic safety in guinea pigs compared to other species, with a 3.80% risk of anaesthetic/sedative fatality reported in guinea pigs in the UK compared with 0.17% for dogs and 0.24% for cats [34]. Differing proportional neutering between the sexes likely reflects the relative ease of surgically neutering males compared to females [35]. Additionally, a single male may be kept with a group of females in a harem [36] in which case neutering the single male rather than all of the females is most efficient. Guinea pigs are social animals, and it is recommended to keep them in single sex pairs, female groups or mixed sex pairs or groups [6]. Low proportional neutering suggests pet guinea pigs are being housed alone or in single sex pairs or groups. In the previously mentioned owner survey, 78.6% of guinea pigs were reportedly housed with a conspecific [6], although as previously stated, those owners may be poorly representative of all owners of guinea pigs in the UK and surveys are also prone to social desirability bias [37]. There are significant welfare implications for guinea pigs housed alone, and owners should be advised accordingly during veterinary consultations [6].

Median bodyweight of male guinea pigs in the current study was significantly heavier than females, consistent with previously reported data [38]. Body condition score was not recorded in our study, so it is not known what proportion of these guinea pigs were above their ideal bodyweight. However, the current results provide a useful benchmark on typical guinea pig bodyweights for veterinary professionals working in primary-care practice.

### Morbidity

The median number of disorders recorded per guinea pigs was one, with no difference identified between males and females. Overall, only 13.13% of guinea pigs under veterinary care had zero disorders recorded, indicating low levels of presentation of healthy pets for routine health checks. This result is similar to that for hamsters presenting to primary-care practice, where 11.93% had no disorder reported [19] and suggests a wide divide in how owners perceive the role of veterinary care for these small exotic species in contrast to more mainstream domestic pet species. For example, similar analyses reported 24.2% of dogs [21], 34.0% of cats [39] and 42.8% of rabbits under primary veterinary care had no disorder recorded [20], likely reflecting their higher level of routine and preventative healthcare (for example neutering, vaccination, microchipping and parasite control). As a prey species, evolutionary survival pressures mean that guinea pigs avoid exhibiting overt signs of illness until disease is advanced [40], so the value of routine health checks should be promoted to owners. The current lack of core vaccinations for guinea pigs prevents their regular presentation for vaccination that offer opportunities for routine health assessment in other companion species such as dogs, cats and rabbits [41]. A UK questionnaire study of guinea pig owners with 4,590 respondents identified that 74.4% of owners only attended a veterinary practice if their guinea pig was sick [6]. These veterinary health checks could also provide opportunities for discussion of husbandry and diet that are linked to several of the more common disorders identified in our study, such as overgrown nails, ocular lesions and bite injuries.

The most common specific disorders recorded among guinea pigs in the current study were overgrown nails and dermatophytosis. Nails grow quickly and may require regular clipping in some individual guinea pigs [1]. Nail overgrowth may indicate underlying husbandry deficits where limited exercise and inappropriate flooring/bedding may lead to reduced nail wear; this may particularly be a problem for guinea pigs housed indoors or in small hutches or bedded on soft fleece bedding, although the competing roles of bedding and flooring for warmth and nail wear do need to be considered [36]. Overgrown nails were also identified as the most common specific disorder diagnosed in rabbits in a study that used a similar study design to the current study [20], indicating that husbandry changes such as alteration to flooring and provision of opportunities for exercise may be required for several small mammalian pets, and this could be discussed during veterinary visits.

Dermatophytosis is widely reported as common in young guinea pigs [1, 42], with one study of pet guinea pigs in Germany reporting a 7.7% dermatophytosis prevalence in guinea pigs with skin lesions, and 8.53% dermatophytosis prevalence in a different sample of asymptomatic guinea pigs sampled for dermatophyte screening [43]. This high frequency of dermatophytosis diagnosis is consistent with our study, where 6.02% of guinea pigs had a positive diagnosis. In contrast, in a study of skin diseases of 293 guinea pigs in the USA, only two cases of dermatophytosis were reported [14]. The guinea pigs in that study were presenting to a specialist exotics clinic and 32% of cases were referrals, which may have biased the results, or this may reflect a true geographical difference in prevalence. Given the consistently high diagnosis in the UK/Europe, clinicians should prioritise testing for dermatophytosis in guinea pigs with skin disease and should alert owners to the zoonotic risk of the disease.

Corneal ulceration was the third most common precise-level disorder, reported in 4.99% of guinea pigs in our study. This high level of ophthalmic disease is consistent with the findings of a prospective study of ophthalmic disease in guinea pigs where 4.7% of animals were diagnosed with conjunctivitis [44]. The relatively high prevalence of corneal ulceration in the current study may be linked to ocular trauma and conjunctival foreign bodies from the natural tendency of guinea pigs to burrow into straw, hay and other substrates [44]. Straw bedding in particular has been implicated as a risk factor for ocular trauma but hay may also present as an ocular traumatic and foreign body risk [16]. Although females were overrepresented for corneal ulceration compared to males in the current study, the reason for this is unclear. Owners should be educated to be vigilant for signs of ocular trauma and questioned regarding husbandry practices when ocular disease cases are diagnosed. Alternative bedding to straw may be advised, alongside avoiding types of hay with a large amount of dust or grass seeds whilst still ensuring access to good quality hay to promote dental wear during chewing. Their preference for burrowing may be facilitated by offering fleecy blankets or soft paper-based bedding to guinea pigs instead of hay or straw, although the competing roles of bedding for comfort, safety, play, nutrition, hygiene and thermal control do need to be considered carefully. This is an area where greater evidence is required. The prevalence of ophthalmic disorders overall was 13.66% in the current study, significantly lower than the 45% previously reported [32]. In that previous prospective study, full ophthalmological examinations were performed in healthy guinea pigs by a veterinary ophthalmologist, so it is unsurprising that a higher prevalence of ophthalmic disorders was detected. Most of those ophthalmic disorders were lens-related changes found in older animals that were only visible on close ophthalmic examination, and therefore less likely to have been noticed by a typical owner to prompt routine primary-care presentation. That study also noted that these age-related lens changes did not appear to have a significant impact on animal health or behaviour. Although close ophthalmic examination should be considered as part of a routine full clinical examination, particularly in older guinea pigs, it is important that owners are advised that many subtle lens related changes are incidental and may have minimal impact on the welfare of their pet.

Dental disease has been reported to be common in guinea pigs, as in other herbivorous small mammals, associated with their entirely elodont dentition [1, 20, 45, 46]. Frequent occurrence in guinea pigs of macrodontia (giant teeth) as on ongoing pathological process characterised by thickening of affected teeth has been proposed as one contributor to a high frequency of dental disease in guinea pigs [47]. Interestingly, at a grouped disorder level, dental disease was diagnosed in just 4.97% of guinea pigs in our study, in contrast to a prevalence of 36.3% reported in guinea pigs presenting to an exotic animal clinic in the Czech Republic [13]. This may reflect a genuinely lower prevalence in the current UK primary-care population or may reflect the differing caseload and clinician experience at an exclusively exotic animal clinic or indeed could reflect international differences such as typical diets offered. A survey of guinea pig owners, distributed via social media and guinea pig forums, reported that 6.7% of 344 guinea pigs were stated by their owners to have a veterinary diagnosis of dental disease despite 16.6% of guinea pigs in that study displaying one or more clinical sign consistent with dental disease (e.g. difficulty eating), suggesting a substantial level of formal veterinary underdiagnosis among the wider general guinea pig population [48]. With an odds ratio of 2.45, males showed a strong predisposition to dental disease compared to females in our study, consistent with the findings of other studies in guinea pigs and in rabbits [13, 20, 47]. Genetic factors feature amongst the suggested aetiologies of dental disease in rodents [49] and may explain the difference between sexes in our study, since environmental and nutritional factors should not differ meaningfully between the sexes. Interestingly, males were also predisposed to anorexia compared to females in our study. Anorexia is a non-specific sign that is commonly associated with dental disease in rodents, so it is possible that further clinical work-up of those guinea pigs with anorexia may have identified undiagnosed underlying dental disease in many cases [45]. The more frequent diagnosis of incisor compared to molar dental disease suggests that many cases of molar dental disease are missed under primary veterinary care, given that incisor malocclusion usually occurs secondary to cheek teeth disorders [11]. Veterinarians should prioritise routine dental examination of guinea pigs, particularly in males, and should educate owners of the clinical signs to monitor to promote early diagnosis and intervention.

Male guinea pigs were significantly more likely to suffer from bite injuries than females in our study. This may reflect higher levels of aggressive and/ or territorial behaviours in male guinea pigs than females, especially when raised as same sex pairs [50]. Information on social groupings was not available in the current study. However, questioning of owners by veterinarians during consultations regarding housing, conspecifics and any behavioural problems observed may help improve husbandry practices and reduce the risk of fighting with conspecifics.

Ovarian disorders accounted for 3.75% of precise level disorders in female guinea pigs in our study, with a median age at diagnosis of 4.62 years. In contrast, a retrospective study of 655 postmortem examinations detected ovarian cysts in 37.4% of entire female guinea pigs [7] suggesting that the results of the current study may be a substantial underestimate. The higher prevalence in this latter pathological study may include identification of incidental ovarian lesions unassociated with clinical signs. A retrospective clinical study reported an overall prevalence of ovarian cysts in pet guinea pigs of 21.9%, with a higher 42.9% prevalence in middle-aged guinea pigs [13]. Ovarian cysts are often detected clinically by abdominal palpation and may not be associated with clinical signs [13]. It is possible the prevalence of cysts in our study was truly low, or that the current results reflect high levels of under-diagnosis. Abdominal palpation of female guinea pigs, in particular in the area around the ovaries, should be prioritised during veterinary physical examinations, especially with advancing age.

### Mortality

The median age at death of guinea pigs overall in this study was 4.03 years, with females living longer (4.58 years) than males (3.74 years). This overall lifespan is slightly lower than the generally reported lifespan of 5–7 years [13, 51] but is similar to the finding of age at death of 4.1 years in an owner survey [6]. However, it is possible that the population of guinea pigs presented for primary veterinary care are not fully representative of the wider guinea pig population in the UK and therefore generalisation to the wider population should be attempted with caution. Awareness of typical age at death of pet guinea pigs can help veterinarians build realistic expectations for owners, especially at the critical points of purchasing a pet in the first place and then when considering end-of-pet-life decisions. Differing age at death between males and females suggests that males may need changes to husbandry and veterinary care earlier than female guinea pigs, for example more frequent health checks and monitoring of quality of life, as is widely recommended for other pet species of advancing age [52]. Cause of death was recorded for over three quarters of deaths (78.08%), with the most common precise-level causes being anorexia and collapse. Anorexia and collapse are clinical presentations that may be associated with a range of underlying pathologies. As a prey species, guinea pigs tend to mask external signs of illness until disease is advanced [16] and it may be that euthanasia was elected without substantial clinical work-up to identify a firm biomedical diagnosis in some of these cases due to an anticipated poor prognosis. The proportion of guinea pigs with ‘disorder not diagnosed’ (21.93%) is three times higher in our study than the 7.3% found in hamsters using a similar study design [19], with both of these values substantially higher than the 0.81% of dogs with ‘disorder not diagnosed’ [21]. In addition, many of the disorder terms such as anorexia (4.04%) and collapsed (1.90%) that were included in the current study also described vague and syndromic conditions that were not conclusive on a specific primary biomedical cause of illness. This may reflect lack of confidence in veterinarians for dealing with small mammalian pets and supports long-standing calls for enhanced undergraduate and postgraduate teaching in exotic animal medicine [53]. The mechanism of death was euthanasia for 73.78% of deaths in the current study, although it is likely that unassisted deaths were underrepresented because the veterinary practice may not have been informed about the demise of many of the guinea pigs that died away from the practice.

Peri-anaesthetic death accounted for 3.38% of all deaths in the study. Anaesthesia does appear to carry relatively high risk for small mammals, with 3.8% of guinea pigs previously reported to have died within 48 hours of anaesthesia [34]. The type of procedure being undertaken (routine vs emergency) was not recorded in our study, but greater understanding of these effects may enable mitigation of some of the risks associated with anaesthesia in guinea pigs.

Lower respiratory tract disorder at a grouped level accounted for 8.32% of guinea pig deaths and was the third most common grouped cause of mortality in the current study, higher than in a previous study where a prevalence of only 4% was found [13]. Bacterial pneumonia, in particular *Bordetella bronchiseptica*, is common in guinea pigs and can be exacerbated by poor husbandry conditions such as inadequate ventilation, inappropriate bedding materials and poor sanitation [1], indicating opportunities for improved owner education.

Neoplasia and haematuria were more common as causes of death in female guinea pigs compared to males in this study. Both disorders had a median age at death of over 5 years. Neoplasia is reportedly uncommon in guinea pigs and usually occurs in animals over 3 years of age [54]. Haematuria has been previously reported to be common in guinea pigs and may be associated with cystitis and urolithiasis, usually occurring in females over 2.5 years of age [55]. Female predisposition is thought to result from their shorter anogenital distance combined with a wider and shorter urethra that increase the risk of ascending infections from enteric bacteria [55, 56]. The increased prevalence of neoplasia and haematuria in older female guinea pigs suggests examination for these should be incorporated into geriatric health checks and owners counselled to look out for typical clinical signs.

### Limitations

This study had some limitations. The clinical data used were sourced from primary-care practices participating in VetCompass and therefore may not generalise fully to all primary-care practices in the UK. The UK primary care practices in the current study typically provide veterinary care for the full spectrum of companion animal species kept in the UK but individual clinicians are likely to vary widely in their experience, expertise and knowledge on guinea pig veterinary care. This variation may impact the precision and accuracy of the diagnoses recorded in the current study. The depth of diagnostic precision reached varied widely across the cases included in the study and reflects the variation typical within primary veterinary care. Owners of guinea pigs presented for UK veterinary primary care are also likely to vary widely in the motivation to achieve and fund full and final diagnoses for all disorders. The current study should therefore be interpreted as an accurate picture of the diagnoses made in UK primary care practice rather than a full and complete analysis of the true disorder profile of guinea pigs in the UK. Specific breed terms were recorded in only 4.46% of guinea pigs and so breed could not be assessed as a factor influencing disorder risk. The bodyweight values reported in the current study reflect the actual bodyweights of these guinea pigs rather than an ideal or optimal bodyweight. Many disorders and causes of death were recorded as ‘disorder not described’ or were described using the predominant clinical sign, for example anorexia, which limit deeper understanding of the true underlying cause. However, the current study aimed to report the spread of diagnosis-making as it truly exists in UK primary care practice and therefore no efforts were made to clean out vague diagnoses to be able to report on just the subset of animals that were more fully clinically worked up. The study included only guinea pigs presented to veterinary practices, so the conclusions may generalise poorly to all pet guinea pigs in the UK.

## Conclusions

This is the largest study to date on guinea pigs under primary veterinary care in the UK. Many of the most common disorders in guinea pigs were husbandry related: overgrown nails, corneal ulceration and obesity. Male guinea pigs had a shorter lifespan and were predisposed to bite injuries and dental disorders. Females were predisposed to ocular disorders and their cause of death was less likely to be specifically diagnosed than males. The data collected in this study can be used to focus veterinary undergraduate and postgraduate education as well as raising awareness to owners on the most common disorders. This study contributed to a limited evidence-base by reporting on general guinea pig health, the more common disorders as well as sex predisposition, the most common causes of death and raises awareness for the need to keep detailed and accurate clinical notes on patients.

## Supporting information

S1 Table VetCompass mapping table linking precise and grouped levels of diagnostic precision for disorders in guinea pigs. (DOCX)

## Abbreviations

CI: confidence interval
EPR: electronic patient record
CPD: continuing professional development
IQR: interquartile range
OR: odds ratio
