# Supplementary material for: Demography, commonly diagnosed disorders and mortality of guinea pigs under primary veterinary care in the UK in 2019—A VetCompass study
Source: PLoS One. 2024 Mar 27;19(3):e0299464. doi: 10.1371/journal.pone.0299464 (PMC10971687; doi:10.1371/journal.pone.0299464)
Supplement: S1 Table — (DOCX) [file pone.0299464.s001.docx]

S1 Table. VetCompass mapping table linking precise and grouped levels of diagnostic precision for disorders in guinea pigs.

| **Disorder at precise-level of diagnostic precision** | **Disorder at grouped-level of diagnostic precision** |
| --- | --- |
| Abdominal distension | Abdominal disease |
| Abdominal hernia | Hernia |
| Abdominal mass | Mass |
| Abdominal pain | Abdominal disease |
| Abnormal gait | Musculoskeletal disorder |
| Abnormal menace response | Brain disorder |
| Abscess | Abscess |
| Acute heart disease | Heart disease |
| Aimless circling | Brain disorder |
| Allergy | Skin disorder |
| Alopecia | Skin disorder |
| Animal bite | Traumatic injury |
| Anorexia | Appetite disoder |
| Ascites | Abdominal disease |
| Ataxia | Spinal cord disorder |
| Aural discharge | Ear disorder |
| Aural mass | Mass |
| Axilla mass | Mass |
| Bacterial skin disorder | Skin disorder |
| Behaviour disorder | Behaviour disorder |
| Bite injury | Traumatic injury |
| Blindness | Ophthalmological disorder |
| Bone neoplasm | Neoplasia |
| Bronchitis | Lower respiratory tract disorder |
| Bruising disoder | Traumatic injury |
| Caecal impaction | Enteropathy |
| Callus | Skin disorder |
| Cardiopulmonary arrest | Heart disease |
| Carpus joint disorder | Musculoskeletal disorder |
| Cataract | Ophthalmological disorder |
| Cheek pouch - neoplasm | Neoplasia |
| Chemosis | Ophthalmological disorder |
| Cheyletiellosis | Parasite infestation |
| Chlamydiosis | Infection - chlamydia |
| Chronic heart disease | Heart disease |
| Chronic kidney failure | Kidney disease |
| Claw injury | Claw/nail disorder |
| Claw/nail disorder | Claw/nail disorder |
| Coccidiosis | Parasite infestation |
| Collapsed | Collapsed |
| Conjunctival mass | Mass |
| Conjunctivitis | Ophthalmological disorder |
| Constipation | Enteropathy |
| Corn on footpad | Skin disorder |
| Corneal disorder | Ophthalmological disorder |
| Corneal lesion | Ophthalmological disorder |
| Corneal ulceration | Ophthalmological disorder |
| Coughing | Upper respiratory tract disorder |
| Crystalluria | Urinary system disorder |
| Cystitis | Urinary system disorder |
| Defaecation abnormal | Enteropathy |
| Dehydration | Dehydration |
| Dental disease | Dental disorder |
| Dermatitis | Skin disorder |
| Developmental dental disease | Dental disorder |
| Diabetes mellitus | Endocrine system disorder |
| Diarrhoea | Enteropathy |
| Disorder not diagnsosed | Disorder not diagnsosed |
| Disorientated | Behaviour disorder |
| Dog bite | Traumatic injury |
| Dorsal mass | Mass |
| Drinking less | Appetite disoder |
| Dysbiosis | Enteropathy |
| Dysphagia | Oral cavity disorder |
| Dyspnoea | Lower respiratory tract disorder |
| Dystocia | Female reproductive disorder |
| Dysuria | Urinary system disorder |
| Ear disorder | Ear disorder |
| Ear injury | Traumatic injury |
| Ear pinna disorder | Ear disorder |
| Enteritis | Enteropathy |
| Enteropathy | Enteropathy |
| Enterotoxaemia | Enteropathy |
| Entropion | Ophthalmological disorder |
| Epistaxis | Upper respiratory tract disorder |
| Exophthalmos | Ophthalmological disorder |
| Eye proptosed | Ophthalmological disorder |
| Eye swelling | Ophthalmological disorder |
| Eyelid mass | Mass |
| Facial neuropathy/nerve paralysis | Peripheral nervous system disorder |
| Faeces abnormal - reduced | Enteropathy |
| Flank mass | Mass |
| Flank neoplasm | Neoplasia |
| Flea infestation | Parasite infestation |
| Flystrike | Parasite infestation |
| Footpad hyperkeratosis | Skin disorder |
| Foreign body | Foreign body |
| Fracture | Traumatic injury |
| Fractured tooth | Dental disorder |
| Gastric dilation-volvulus syndrome | Enteropathy |
| Gastric disorder | Enteropathy |
| Gastroenteritis | Enteropathy |
| Gastrointestinal stasis | Enteropathy |
| Giardiasis | Enteropathy |
| Glucosuria | Endocrine system disorder |
| Gut stasis | Enteropathy |
| Haematoma | Traumatic injury |
| Haematuria | Urinary system disorder |
| Haemorrhage | Haematopoietic disorder |
| Haircoat disoder | Skin disorder |
| Head tilt | Brain disorder |
| Hearing impaired | Hearing impaired |
| Heart disease | Heart disease |
| Heat stroke | Collapsed |
| Hindlimb mass | Mass |
| Hyperadrenocorticism | Endocrine system disorder |
| Hyperkeratosis | Skin disorder |
| Hypoglycaemia | Lethargy |
| Hypothermia | Lethargy |
| Ileus | Enteropathy |
| Inappetance | Appetite disoder |
| Increased salivation | Oral cavity disorder |
| Inflammation of lips | Oral cavity disorder |
| Interdigital mass | Mass |
| Intestinal mass | Mass |
| Intestinal obstruction | Enteropathy |
| Intra-ocular mass | Mass |
| Jaw disorder | Oral cavity disorder |
| Jaw mass | Mass |
| Joint mass/swelling | Musculoskeletal disorder |
| Kennel Cough | Upper respiratory tract disorder |
| Keratoconjunctivitis sicca | Ophthalmological disorder |
| Kidney disorder | Kidney disease |
| Kidney failure | Kidney disease |
| Kidney mass | Mass |
| Laceration | Traumatic injury |
| Lameness | Musculoskeletal disorder |
| Lameness finding | Lameness finding |
| Lethargy | Lethargy |
| Leukaemia | Neoplasia |
| Lice | Parasite infestation |
| Limb mass | Mass |
| Limb neoplasm | Neoplasia |
| Limb swelling | Musculoskeletal disorder |
| Limb weakness | Musculoskeletal disorder |
| Lip mass | Mass |
| Lip neoplasm | Neoplasia |
| Lipoma | Neoplasia |
| Liver disorder | Liver disorder |
| Lower respiratory tract infection | Lower respiratory tract disorder |
| Lung disorder | Lower respiratory tract disorder |
| Lung neoplasm | Neoplasia |
| Lymphadenitis | Lymph node disorder |
| Lymphadenopathy | Lymph node disorder |
| Lymphoma | Neoplasia |
| Mammary mass | Mass |
| Mammary neoplasm | Neoplasia |
| Mange | Parasite infestation |
| Mass | Mass |
| Mass lesion - chin | Mass |
| Mast cell tumour | Neoplasia |
| Meibomian cyst | Ophthalmological disorder |
| Microphthalmos | Ophthalmological disorder |
| Miscarriage - Abortion | Female reproductive disorder |
| Mite infestation | Parasite infestation |
| Moist dermatitis | Skin disorder |
| Moribund | Collapsed |
| Multiple masss | Mass |
| Muscle atrophy | Musculoskeletal disorder |
| Musculoskeletal disorder | Musculoskeletal disorder |
| Musculoskeletal injury | Musculoskeletal disorder |
| Musculoskeletal pain | Musculoskeletal disorder |
| Nasal discharge | Upper respiratory tract disorder |
| Nasal mass | Mass |
| Neck mass | Mass |
| Neck neoplasm | Neoplasia |
| Neoplasia | Neoplasia |
| Neurological disorder | Brain disorder |
| Neuromuscular disorder | Peripheral nervous system disorder |
| Normal | Normal |
| Obesity | Obesity |
| Ocular discharge | Ophthalmological disorder |
| Ophthalmic injury | Ophthalmological disorder |
| Ophthalmological disorder | Ophthalmological disorder |
| Oral cavity disorder | Oral cavity disorder |
| Osteoarthritis | Musculoskeletal disorder |
| Osteomyelitis | Musculoskeletal disorder |
| Otitis externa | Ear disorder |
| Otitis interna | Ear disorder |
| Otitis media | Ear disorder |
| Ovarian disorder | Female reproductive disorder |
| Ovarian mass | Mass |
| Ovarian neoplasm | Neoplasia |
| Overgrooming | Skin disorder |
| Overgrown incisor(s) | Dental disorder |
| Overgrown molar(s) | Dental disorder |
| Overgrown nail(s) | Claw/nail disorder |
| Pain | Lethargy |
| Parasite infestation | Parasite infestation |
| Paresis/paralysis | Spinal cord disorder |
| Paronychia | Claw/nail disorder |
| Pelvic limb fracture | Traumatic injury |
| Penile mass | Mass |
| Peri-anaesthetic death | Complication associated with clinical care |
| Perineal faecal impaction | Skin disorder |
| Periodontal disease | Dental disorder |
| Peripheral oedema | Heart disease |
| Pleural effusion | Lower respiratory tract disorder |
| Pneumonia | Lower respiratory tract disorder |
| Pododermatitis | Skin disorder |
| Polydactyly | Congenital disorder |
| Polyuria/polydipsia | Polyuria/polydipsia |
| Poor quality of life | Poor quality of life |
| Post-operative wound | Complication associated with clinical care |
| Proprioception deficits | Neurological disorder |
| Protozoal infection | Protozoal infection |
| Pruritus | Skin disorder |
| Pyelonephritis | Kidney disease |
| Pyoderma | Skin disorder |
| Pyometra | Female reproductive disorder |
| Rectal disorder | Enteropathy |
| Respiratory distress | Lower respiratory tract disorder |
| Respiratory noise increased | Lower respiratory tract disorder |
| Respiratory system disorder | Lower respiratory tract disorder |
| Retrobulbar mass | Mass |
| Ringworm | Skin disorder |
| Scab lesion(s) | Skin disorder |
| Scabies | Parasite infestation |
| Scaling dermatosis | Skin disorder |
| Seizure disorder | Brain disorder |
| Shock | Shock |
| Skin cyst | Neoplasia |
| Skin disorder | Skin disorder |
| Skin fold dermatitis | Skin disorder |
| Skin lesions | Skin disorder |
| Skin mass | Mass |
| Sneezing | Upper respiratory tract disorder |
| Soft tissue injury | Soft tissue disorder |
| Spinal cord injury | Spinal cord disorder |
| Spinal pain | Spinal cord disorder |
| Stroke | Brain disorder |
| Subcutaneous tissue mass | Mass |
| Thin | Thin |
| Thoracic mass | Mass |
| Thoracic wall mass | Mass |
| Thoracic wall neoplasm | Neoplasia |
| Tongue mass | Mass |
| Torn nail | Claw/nail disorder |
| Traumatic injury | Traumatic injury |
| Tremors/shaking/trembling | Brain disorder |
| Trichoblastoma | Neoplasia |
| Ulceration disorder | Skin disorder |
| Umbilical hernia | Hernia |
| Upper respiratory tract disorder | Upper respiratory tract disorder |
| Upper respiratory tract infection | Upper respiratory tract disorder |
| Urethral obstruction | Urinary system disorder |
| Urinary bladder - calculus | Urinary system disorder |
| Urinary bladder neoplasm | Neoplasia |
| Urinary system disorder | Urinary system disorder |
| Urinary tract infection | Urinary system disorder |
| Urolithiasis | Urinary system disorder |
| Uterine prolapse | Female reproductive disorder |
| Uveitis | Ophthalmological disorder |
| Vaginal hyperplasia/prolapse | Female reproductive disorder |
| Vaginal/vulval discharge | Female reproductive disorder |
| Weight loss | Thin |
| Wound | Traumatic injury |
| Penile/prepuce disorder | Male reproductive system disorder |
| Penile/prepuce disorder | Penile/prepuce disorder |
